# Primary Nasal Tuberculosis in a 10-Year-Old Girl

**DOI:** 10.1155/2016/9128548

**Published:** 2016-03-29

**Authors:** Murat Özer, Yasemin Özsurekçi, Ali Bülent Cengiz, Uğur Özçelik, Ebru Yalçın, Özay Gököz

**Affiliations:** ^1^Department of Pediatrics, Hacettepe University Faculty of Medicine, 6100 Ankara, Turkey; ^2^Pediatric Infectious Diseases, Hacettepe University Faculty of Medicine, 6100 Ankara, Turkey; ^3^Pediatric Chest Diseases, Hacettepe University Faculty of Medicine, 6100 Ankara, Turkey; ^4^Department of Pathology, Hacettepe University Faculty of Medicine, 6100 Ankara, Turkey

## Abstract

Nasal tuberculosis is a rare clinical entity which mainly presents in elderly people. Nasal tuberculosis has always been considered to be secondary to tuberculosis of the lungs, and in rare instances it is a primary infection, usually when mycobacteria are inhaled. We describe the case of a 10-year-old girl who was successfully treated for primary nasal tuberculosis. This patient is one of the very few children who have been reported to have primary nasal tuberculosis.

## 1. Introduction

Nasal tuberculosis was first described by an Italian anatomist in 1761, when a young man's autopsy had found an ulcerative lesion on the nose [1]. Nasal tuberculosis had always been considered to be secondary to tuberculosis of the lungs, and it rarely presents as a local disease in the form of a primary infection [2]. Primary nasal tuberculosis is a rare clinical entity, and only 35 cases were reported in a review of the 20th century medical literature, which was published in 1997, and the diagnoses were mostly in elderly patients [3]. It is difficult to determine the presence of nasal tuberculosis, because it shows subtle signs and symptoms [4]. The common presenting symptoms of nasal tuberculosis are ulcerative lesions within the nasal cavity, nasal obstruction, nasal discharge, epistaxis, crusting, recurrent nasal polyps, and other ulceration [5].

According to the World Health Organization's data, the annual incidence of tuberculosis in Turkey was 18/100,000 for the year 2014. It was also reported that 4% of both first-time and relapsing TB sufferers were under 15 years of age [6]. Herein, we describe a 10-year-old girl with primary nasal tuberculosis, who presented with a persistent ulcerative nasal lesion. The patient was successfully treated with antituberculosis therapy without any complications.

## 2. Case Report

A 10-year-old girl had crusts and persistent wounds on the nose and in the internal mucosal layer for six months. She also had progressive nasal stuffiness. She had not received any medication. There were no known diseases in her previous history. Her father was treated for pulmonary tuberculosis 20 years before this presentation; however, there were no active complaints in his clinical follow-up, and no other family members had any history of tuberculosis. There were also no close blood-relatives who had been diagnosed with tuberculosis. It was learned from the family that a bacille Calmette-Guerin (BCG) vaccination was given at 2 months of age based on the vaccination schedule in Turkey.

**Figure 1:**
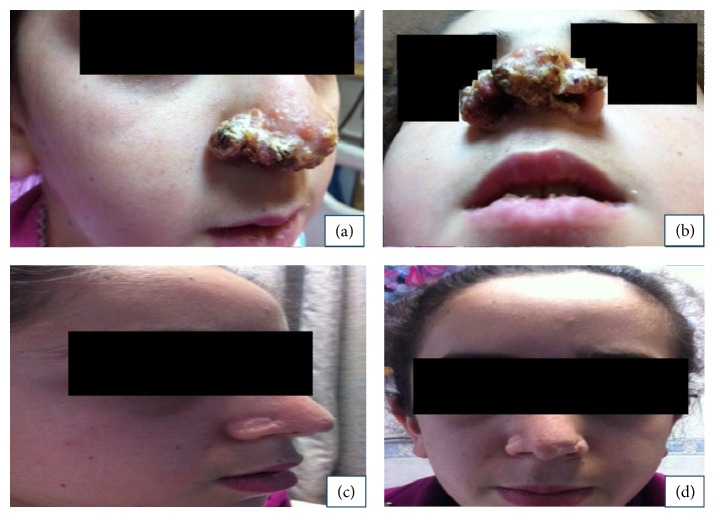
A reddish ulcerative lesion on the nose and in the internal mucosal layer at the admission: (a) right side and (b) front side. Improvement of the lesion 2 years later: (c) right side and (d) front side.

On physical examination, her body weight was 25 kg (25–50th percentile), and her height was 115 cm (50th percentile). There were erythematous, edematous, yellow-brown colored, and thick-crusted plaques on her nose (Figures 1(a) and 1(b)). There were no systemic symptoms.

**Figure 2:**
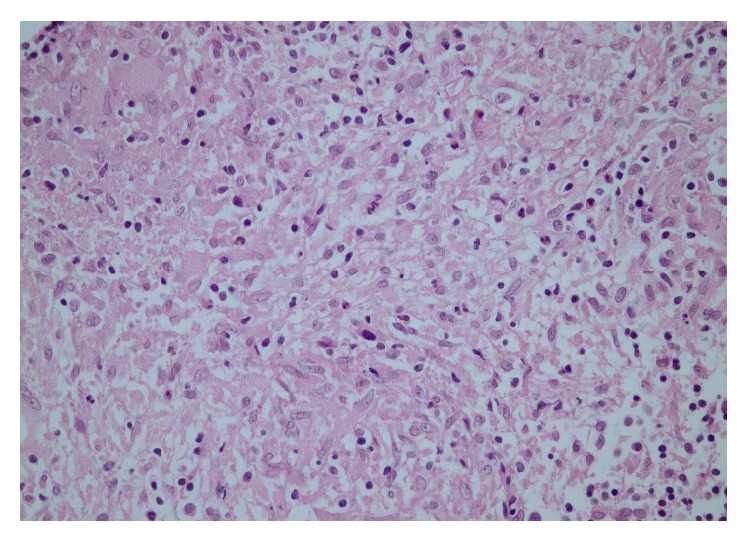
Occasional giant cells and inconspicuous necrosis at the center. HE ×400.

The patient was hospitalized for investigation. Her laboratory tests, which included a complete blood count, peripheral smear, and tests for kidney and liver function, were within normal limits. The erythrocyte sedimentation rate was 57 mm/h (0–20), and the C-reactive protein was 0.41 mg/dL (0–0.8). The Venereal Disease Research Laboratory (VDRL) and Human Immunodeficiency Virus (HIV) antibody tests were negative. Imaging studies were taken of the patient's lungs with no abnormality found. The purified protein derivative (Mantoux test) was strongly positive, with a maximum diameter of 24 mm of induration. A punch biopsy of the lesion was performed: histology revealed pseudoepitheliomatous hyperplasia, as well as a subepithelial lymphocytic reaction of histocytes and giant cells with a granulomatous reaction in the epidermis (Figure 2). No specific agent was shown on acid-resistant bacilli (ARB) or Giemsa and Gomori's Methenamine Silver (GMS) stains.* M. tuberculosis* was isolated from the tissue culture. The isolate was resistant to streptomycin and ethambutol and sensitive to isoniazid and rifampicin. The patient was assessed to have primary tuberculosis with nasal involvement. The induration diameters of PPD were detected to be 4 mm and 3 mm in the mother and sister, respectively. A chest radiograph was normal. Additional images from a contrast-enhanced computerized tomography (CT) scan of the head demonstrated that the nasal septum was normal. Isoniazid (10 mg/kg/day), rifampicin (10 mg/kg/day), and pyrazinamide (30 mg/kg/day) treatments were started. A primary immunologic workup of serum quantitative immunoglobulin, lymphocyte subsets, and nitroblue tetrazolium (NBT) levels was normal.

The patient was discharged after 15 days of hospitalization. Two months later, she came for an outpatient clinic visit with signs of improvement. At the end of the second month, pyrazinamide was discontinued. Six months later, isoniazid and rifampicin treatments were also discontinued. There were no interruptions in drug administration during the treatment period, and no drug complications were observed. The total treatment plan was completed in 6 months. The skin lesion had almost completely resolved 24 months after her first admission to our department (Figures 1(c) and 1(d)).

## 3. Discussion

Nasal tuberculosis can present in a spontaneous form, secondarily after pulmonary tuberculosis, or in a primary form, without prior pulmonary infection. It was reported that spontaneous nasal tuberculosis was more common than primary nasal tuberculosis [10]. Nasal tuberculosis is generally reported to occur in people between 20 and 84 years of age and is more common in women than men [3]. However, the number of pediatric cases in the literature is limited; these include an 11-year-old male and a 10-year-old female from India, as well as a 12-year-old female from England [7–9]. Our case is one of the very few published pediatric cases, and we wanted to emphasize the importance of this rare childhood presentation of tuberculosis.

Although the major symptom of the three reported cases was epistaxis [7–9], the predominant symptom of our case was nasal congestion. There was no lymphadenopathy, or any other tuberculous foci identified on physical examination. There were no signs of pulmonary tuberculosis on the chest radiograph or scans. Accordingly, the patient was considered to have primary nasal tuberculosis.* M. tuberculosis* may be introduced into the nasal cavity either through local infiltration by finger contact or by inhaling infected droplets or dust [5]. We may speculate that our patient contracted the infection via direct inoculation.

Stuffiness (nasal obstruction), epistaxis, nasal discharge, incrustation, recurrent nasal polyps, and ulcers are the primary symptoms of nasal tuberculosis. The lesions can be ulcerative, infiltrative, or proliferative and in most cases are unilateral [10]. The formation of nodules on anatrophic- or cicatrice-formed lesion is one characteristic finding of nasal tuberculosis [5] and was noted in our patient's case. The most common site of involvement in the nose is the nasal septum, and nasal perforation is one of its complications [4]. A nasal computed tomography scan was performed in our patient to locate any abnormalities or perforations of the nasal septum, and the images were normal.

Diagnosing nasal tuberculosis is difficult because of the nonspecific symptoms. A definitive diagnosis can be made by biopsy and isolation of the tubercle bacillus from surgically removed tissue [11]. However, the culture results may be negative in some cases because of a very low number of mycobacteria in the tissue. Thus, it is important to have histopathological examinations of skin lesions [11]. A granulomatous reaction was revealed in the punch biopsy of our patient as well as in the isolation of* M. tuberculosis* from the tissue culture, which confirmed the diagnosis. Isolation of the pathogen is important because other mycobacterial and nonmycobacterial agents can cause granulomatous inflammation.

Nasal tuberculosis should be treated according to the established general guidelines for extrapulmonary tuberculosis, although it may be necessary to choose alternative medications based on the drugs' availability and the resistance patterns of the strains. Three or more drugs are used for a short initial bactericidal regimen, which is followed by a prolonged regimen that uses at least 2 drugs [12]. Isoniazid, rifampicin, and pyrazinamide were administered for 2 months in a triple antituberculosis treatment plan for our patient. Isoniazid and rifampicin were continued for 4 months afterwards with a good clinical response. If there are no complications, surgical treatment is not necessary in localized nasal disease. Standard antituberculosis therapy is usually enough to completely cure the disease [13]. There were no complications in our patient, so surgical treatment was not needed.

The possibility of nasal tuberculosis should be kept in mind during the differential diagnosis of patients with chronic nasal lesions, particularly those who are the children and residents of countries more highly endemic for tuberculosis such as Turkey.
